# Intussusception caused by an inverted Meckel's diverticulum: a rare cause of small bowel obstruction in adults

**Published:** 2011-12-18

**Authors:** Mahdi Bouassida, Bilel Feidi, Mechaal Ben Ali, Mohamed Fadhel Chtourou, Marouene Krifa, Selim Sassi, Fathi Chebbi, Mohamed Mongi Mighri, Hassen Touinsi, Sadok Sassi

**Affiliations:** 1 Department of surgery, Mohamed Thahar Maamouri Hospital, Nabeul, Tunisia; 2 Department of reanimation, Mohamed Thahar Maamouri Hospital, Nabeul, Tunisia

**Keywords:** Meckel diverticulum, intussusception, inverted diverticulum, emergency surgery, intestinal obstruction

## Abstract

Adult intussusception due to Meckel's diverticulum is an uncommon cause of intestinal obstruction. However, the surgeon should still be suspicious of this condition since the non specific symptoms and the rarity of it make a preoperative diagnosis uncertain. Considering the secondary nature of adult intussusception and the necessity of early surgical intervention to avoid morbidity and mortality, we report one case of intussusception due to Meckel's diverticulum in an adult. A 22-year-old patient was admitted to our hospital with vomiting and abdominal pain. The abdomen was hard with tenderness. We diagnosed an acute small bowel obstruction and performed emergency surgery. The intra operative findings were distention of the small bowel and intussusception of ileus due to an inverted Meckel's diverticulum located 70 cm from the ileocecal valve. 30 cm ischemic loop was identified. A segmental small bowel resection and hand-sewn anastomosis was performed. Histopathology distinguished Meckel's diverticulum measuring 5 cm x 3.5 cm x 1 cm and no signs of malignancy.

## Introduction

Meckel's diverticulum is the most common congenital abnormality of the gastrointestinal tract, occurring in 1% to 2% of the population. It is usually asymptomatic and becomes evident when complicated. Intestinal obstruction due to Meckel′s diverticulum is the most common presentation in adult and is the second most common in children. Occasionally, inversion of Meckel's diverticulum into the lumen of the bowel can cause intussusception, ischemia and infarction. The incidence of intussesception attributed to an inversion of Meckel's diverticulum accounts for 4% of all cases presenting with intestinal obstruction due to intussusception.

It occurs when the Meckel's diverticulum sags into the bowel lumen and then serves as a lead point to allow telescoping of the small intestine, first into the distal ileum and then in to the large intestine, causing ileo-ileal and ileocolic type of intussusceptions.

## Case report

**Figure 1:**
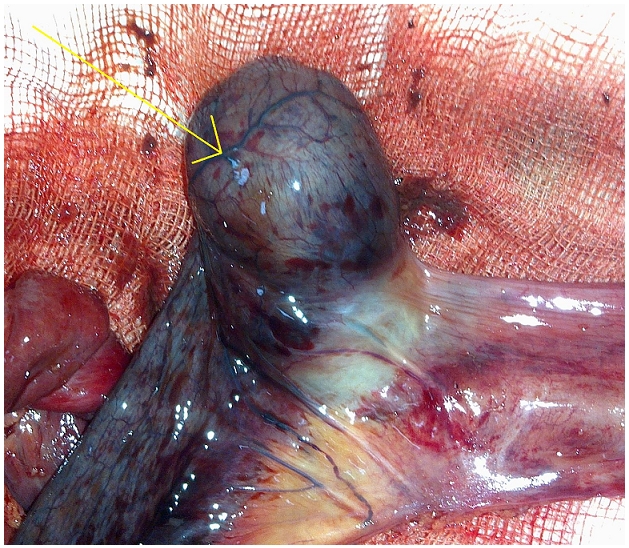
The surgical specimen after reduction comprised a 30 cm segment of resected ileus with an elongated polypoid lesion (arrow) within the ileal lumen, and a lengthy segment of intestine was necrotic

A 22-year-old male presented to the emergency room complaining of abdominal pain and vomiting for one day. He had no significant past medical history or previous abdominal surgery. There was no family history of any hereditary illnesses. On admission he had normal vital signs and a temperature of 38°C. Physical examination showed a slightly distended abdomen with hyperactive bowel sounds. The patient had abdominal tenderness and muscle guarding. No masses or hernias were identified. Laboratory tests revealed increased white blood cells (WBC) of 13 200 µ/L. Plain abdominal X-ray demonstrated air fluid levels of the small bowel, there was no pneumoperitomeum. We diagnosed an acute small bowel obstruction. Because of severe abdominal pain, the patient underwent emergency surgery with a lower midline abdominal incision. The intra operative findings were distention of the small bowel and intussusception of ileus due to an inverted Meckel's diverticulum located 70 cm from the ileocecal valve. 30 cm ischemic loop was identified (Figure 1). A segmental small bowel resection and hand-sewn anastomosis was performed.

Histopathology revealed Meckel's diverticulum measuring 5 cm x 3.5 cm x 1 cm without ectopic mucosa or malignancy. The postoperative period was uneventful and after 7 days the patient was discharged. At the 6 months follow-up, the patient had no evidence of complications.

## Discussion

Intestinal obstruction due to Meckel′s diverticulum is the most common presentation in adult and is the second most common in children [1]. There are various mechanisms by which it can cause intestinal obstruction like (a) Volvulus of small intestine around a fibrous band extending from Meckel′s diverticulum to umbilicus. (b) Intussusception – in which Meckel's diverticulum sags into the bowel lumen and then serves as a lead point to allow telescoping of the small intestine into first the distal ileum and then in to the large intestine causing ileo-ileal and ileocolic type of intussusception. (c) Littre′s hernia – Incarceration of the diverticulum in hernia, (inguinal and femoral) causing intestinal obstruction. (d) Entrapment of small bowel beneath the blood supply of the diverticulum, also known as a mesodiverticular band (e) Stricture secondary to chronic diverticulitis [2] (f) Meckel′s diverticulum lithiasis (g) Band extending between the diverticulum and the base of the mesentery, forming a loop in which a part of ileum may get stuck causing obstruction [3]. Inversion of Meckel's diverticulum is not yet clearly understood. One theory is that abnormal peristaltic movement due to ulceration or ectopic tissue at the base of Meckel's diverticulum may cause it to invert [4]. The presenting symptoms in adult patients with intussusceptions are nonspecific and often long-standing. The most important characteristic of pain is its periodic, intermittent nature, which makes the diagnosis elusive and accounts for the delay in establishing the diagnosis [5]. CT is the most sensitive imaging modality for diagnosis of bowel obstruction with reported accuracy of 58%-100% [6]. In the early stage, the characteristic point is the target lesion, described as enveloped, eccentrically located areas of low density, which represents the intussuscepted bowel viewed in cross section [7]. Other findings, such as vascular compromise, layering or stratification effect to bowel wall thickening and amorphous mass are recognized later in the natural history of unrelieved intussusceptions [8].

Intra operatively, the intussuscepted bowel should be deployed and examined for ischemia. There is no doubt that in case of transmural ischemia, the bowel needs to be resected along with the diverticulum [9]. Intussusception due to Meckel's diverticulum is an absolute indication for Meckel's diverticulum resection [8]. Recently, the prevalence of laparoscopic surgery has been increasing. However, the procedure should be chosen carefully because of an increased risk of perforation [8]. In our case, the abdomen was hard with tenderness and muscle guarding, and the leukocyte count was increased. These findings suggested an intussusceptions caused by benign lesion, but we did not choose a laparoscopic procedure because we considered that a lengthy segment of intestine was necrotic.

## Conclusion

Adult intussusception caused by inverted Meckel's diverticulum may be observed in any age. It is a rare but important clinical entity. The presenting symptoms of intussusception are nonspecific. Diverticulectomy or bowel resection is the standard treatment.
